# *In vitro* anti-*Toxoplasma gondii* efficacy of synthesised benzyltriazole derivatives

**DOI:** 10.4102/ojvr.v88i1.1898

**Published:** 2021-06-11

**Authors:** Huanping Guo, Yang Gao, David D. N’Da, Xuenan Xuan

**Affiliations:** 1 National Research Center for Protozoan Diseases, Obihiro University of Agriculture and Veterinary Medicine, Obihiro, Japan; 2 Centre of Excellence for Pharmaceutical Sciences, Faculty of Health Sciences, North-West University, Potchefstroom, South Africa

**Keywords:** toxoplasmosis, tachyzoite, bradyzoite, benzyltriazole, anti-*Toxoplasma gondii* efficacy, *in vitro*

## Abstract

*Toxoplasma gondii*, an obligate intracellular parasite, is the aetiological agent of toxoplasmosis, a disease that affects approximately 25% – 30% of the world’s population. At present, no safe and effective vaccine exists for the prevention of toxoplasmosis. Current treatment options for toxoplasmosis are active only against tachyzoites and may also cause bone marrow toxicity. To contribute to the global search for novel agents for the treatment of toxoplasmosis, we herein report the *in vitro* activities of previously synthesised benzyltriazole derivatives. The effects of these compounds against *T. gondii in vitro* were evaluated by using a expressing green fluorescent protein (GFP) type I strain parasite (RH-GFP) and a type II cyst-forming strain of parasite (PruΔku80Δhxgprt). The frontline antitubercular drug isoniazid, designated as Frans J. Smit -isoniazid (FJS-INH), was also included in the screening as a preliminary test in view of future repurposing of this agent. Of the compounds screened, FJS-302, FJS-303, FJS-403 and FJS-INH demonstrated > 80% parasite growth inhibition with IC_50_ values of 5.6 µg/mL, 6.8 µg/µL, 7.0 µg/mL and 19.8 µg/mL, respectively. FJS-302, FJS-303 and FJS-403 inhibited parasite invasion and replication, whereas, sulphadiazine (SFZ), the positive control, was only effective against parasite replication. In addition, SFZ induced bradyzoite differentiation *in vitro*, whilst FJS-302, FJS-303 and FJS-403 did not increase the bradyzoite number. These results indicate that FJS-302, FJS-303 and FJS-403 have the potential to act as a viable source of antiparasitic therapeutic agents.

## Introduction

The protozoan parasite *Toxoplasma gondii* is an obligate intracellular pathogen, which belongs to the phylum Apicomplexa, and virtually infects any kind of warm-blooded animal, including humans (Dubey 2010). Approximately 25% – 30% of the world’s population is infected with *T. gondii* (Maenz et al. 2014). The life cycle of *T. gondii* includes a sexual stage that develops only in definitive hosts such as cat and an asexual stage, which can develop in definitive and intermediate hosts including various warm-blooded animals (Dubey 2010). Asexual reproduction occurs in two phases: firstly, tachyzoites (or endozoites) replicate rapidly by repeated endodyogeny in host cells, and then secondly the next phase starts from the last generation of the tachyzoites that transform into tissue cysts in which bradyzoites (or cystozoites) replicate slowly by endodyogeny (Dubey & Beattie 1998; Dubey, Lindsay & Speer 1998). The tachyzoite can cause a strong inflammatory response and tissue destruction and therefore is responsible for clinical manifestations of toxoplasmosis. On the other hand, bradyzoites persist inside cysts for the lifetime of the host (Maenz et al. 2014), and in immunocompromised patients bradyzoites can be released from cysts, transformed back into tachyzoites and can cause reactivation of the infection (Weiss & Kim 2000).

Primary infection with *T. gondii* in pregnant women or animals can lead to congenital diseases such as hydrocephalus and chorioretinitis in newborn children (Goldstein, Montoya & Remington 2008). Currently, there is no safe and effective vaccine for preventing toxoplasmosis. An effective chemotherapy constitutes the only alternative to control the disease. Medications expressing antibacterial (sulphadiazine [SFZ], clindamycin and spiramycin) or antimalarial activity (pyrimethamine [PYR] and atovaquone) are the classical chemotherapy (Antczak, Dzitko & Długońska 2016). It is noteworthy that the therapy of the disease based on these drugs is active only against tachyzoites and limited in eliminating encysted bradyzoites (McLeod et al. 2006). Moreover, the recommended chemotherapy involves a combination of SFZ and PYR. However, synergistic action of SFZ and PYR disturbs folic acid biosynthesis and is also toxic to human cells (Antczak et al. 2016). Therefore, novel efficacious drugs for toxoplasmosis are urgently needed.

The triazole core, a well-known privileged structure, has drawn much attention in drug discovery, including a five-member *N*-heterocyclic compound 1,2,3-triazole (Ali et al. 2017; Antczak et al. 2016; Dheer et al. 2017). Many compounds containing 1,2,3-triazole have showed good activities in many different diseases, such as antitubercular (TB) (Boechat et al. 2011), antifungal (Dai et al. 2015), antihuman immunodeficiency virus (HIV) (Mohammed et al. 2016), antimalarial (Kumar et al. 2014a; Singh et al. 2017), anti-inflammatory (Shafi et al. 2012) and antitoxoplasmosis activities (Alday & Doggett 2017; Dzitko et al. 2014; Luan et al. 2019). The moiety possesses hydrogen-bonding capability, moderate dipole character, rigidity and stability under *in vivo* conditions, which all together are responsible for its enhanced biological properties (Zhang et al. 2017). Interestingly, some compounds containing triazole and isoniazid share a similar mechanism of action as they inhibit microbial cell wall synthesis by blocking lipid biosynthesis (Kumar et al. 2014b; Zhang et al. 2017).

In an effort to contribute to the global search for effective and safe new agents for the treatment of toxoplasmosis, we herein report the *in vitro* anti-*T. gondii* efficacy of previously synthesised benzyltriazole (BnTz) derivatives.

## Materials and methods

### Cytotoxicity analysis

The cytotoxicity of the chemical compounds on human foreskin fibroblasts (HFF) cells was determined by using the cell counting kit-8 (CCK-8) (Dojindo Molecular Technologies, Inc. Japan) according to the manufacturer’s instructions. The compounds were dissolved in dimethyl sulfoxide (DMSO). HFF cells were plated in 96-well plates at a density of 1 × 10^4^ cells per well. After a 48-h incubation at 37 °C in a 5% CO_2_ atmosphere, cells were exposed to various concentrations of tested chemical compounds (1 µg/mL, 5 µg/mL, 10 µg/mL, 25 µg/mL, 50 µg/mL and 100 µg/mL). Culture medium containing the same volume of DMSO was used as a negative control. After 24 h, the surviving cells were determined by adding CCK-8 reagent. Cell viability was measured based on the absorbance at 450 nm after an additional 4-h incubation.

### *In vitro* growth inhibition assay

To evaluate the activities of synthesised chemical compounds and FJS-INH on *T. gondii in vitro*, a preliminary screening was performed at a single concentration of 50 µg/mL as previously described (Leesombun et al. 2016). Briefly, HFF cells were seeded into 96-well plates (1 × 10^4^ cells/well) and cultured for 48 h. Expressing green fluorescent protein (GFP) type I strain RH (RH-GFP) tachyzoites (Nishikawa et al. 2003) were added to the wells (5 × 10^4^ tachyzoites/well). After a 4-h incubation, extracellular parasites were removed by washing. Then, chemical compounds at a final concentration of 50 µg/mL were added. Medium and SFZ (1 mg/mL)-treated infected wells were used as negative and positive controls, respectively, whilst uninfected wells treated with ‘medium’ or ‘medium’ or ‘compounds’ were used to correct for background signal. After a 72-h incubation, the fluorescence intensity of RH-GFP was measured to determine the parasite growth. Compounds with parasite inhibition ≥ 80% were further screened for dose–response effects at final concentrations of 3.125 µg/mL, 6.25 µg/mL, 12.5 µg/mL, 25 µg/mL and 50 µg/mL.

### Effects of chemical compounds on *Toxoplasma gondii* replication *in vitro*

To evaluate effect of the screened chemical compounds (FJS-302, FJS-303, FJS-403 and FJS-INH) on parasite replication, Vero cells were plated in 12-well plates at a density of 1 × 10^5^ cells per well and incubated for 24 h at 37 °C in a 5% CO_2_ atmosphere as previously described. RH-GFP tachyzoites were then added to Vero cells at 1 mL/well (parasites per host cell ratio = 2:1). At 2 h post-infection, the extracellular parasites were removed by washing, and chemical compounds were added (FJS-302, 10 µg/mL; FJS-303, 12.5 µg/mL; FJS-403, 12.5 µg/mL; FJS-INH, 50 µg/mL or 1 mg/mL of SFZ) in Eagle’s minimum essential medium (Sigma, United States (US)) (EMEM) (Sigma, United States of America) supplemented with 8% foetal bovine serum (FBS) (Biowest, Japan). After a 24-h incubation, indirect fluorescent antibody test (IFAT) was performed as previously described (Leesombun et al. 2016) by using anti-surface antigen 1 (SAG1) mouse polyclonal antibody (Guo et al. 2019). Parasite replication in Vero cells was determined by counting the number of tachyzoites per parasitophorous vacuole (PV) (at least 100 vacuoles were randomly selected per well).

### Effects of chemical compounds on *Toxoplasma gondii* invasion *in vitro*

Vero cells were seeded on 12-well plates as described here, and purified RH-GFP tachyzoites were treated with FJS-302, FJS-303, FJS-403 or FJS-INH for 1 h at 37 °C. Then treated parasites were added to Vero cells in a 12-well plate (2 × 10^5^ tachyzoites per well). At 2 h postinfection, the extracellular parasites were removed by washing, and the infected Vero cells were further incubated for 24 h at 37 °C in a 5% CO_2_ incubator. After incubation, IFAT was conducted as described here to evaluate the effects of the chemical compounds on parasite invasion. The infection rates were calculated as follows:

$$\frac{\left[\text{number of SAG1-positive Vero cells}\right]}{\left[100\text{ randomly selected Vero cells}\right]}\times 100$$ [Eqn 1]

In addition, at least 10 fields were observed per group to measure the average number of parasites per field.

### Bradyzoite differentiation assay

To evaluate the effect of FJS-302, FJS-303, FJS-403 and FJS-INH on spontaneously induced bradyzoite differentiation *in vitro*, PruΔku80Δhxgprt (Fox et al. 2011; Murata et al. 2017) that expresses GFP under the control of bradyzoite-specific gene lactate dehydrogenase 2 (LDH2) promoter was used. Vero cells were plated in 12-well plates at a density of 1 × 10^5^ cells per well and incubated for 24 h at 37 °C in a 5% CO_2_ atmosphere. Then, purified PruΔku80Δhxgprt tachyzoites were added to Vero cells at 1 mL/well (parasites per host cell ratio = 1:1). Two hours postinfection, chemical compounds were added in EMEM supplemented with 8% FBS. Indirect fluorescent antibody test was conducted after a 48-h incubation. Tachyzoites were stained by using anti-SAG1 mouse polyclonal antibody (Guo et al. 2019), and bradyzoites were detected by using anti-GFP rabbit polyclonal antibody (Medical & Biological Laboratories CO., LDT, Japan). Secondary antibody Alexa Fluor 594-conjugated goat anti-mouse IgG (Invitrogen, United States) diluted 1:1000 and Alexa Fluor 488-conjugated goat anti-Rabbit IgG (Invitrogen, United States) diluted 1:1000 were used. Five fields per well were observed by using an All-in-one Fluorescence Microscope (BZ-9000, Keyence, Japan), and the percentage of bradyzoites was calculated.

### Statistical analysis

Statistical analyses were performed by using one-way analysis of variance (ANOVA) followed by the Tukey-Kramer test for group comparisons. Data were expressed as the mean ± standard deviation (s.d.). All data were analysed by using GraphPad Prism 8 software (GraphPad Software Inc., United States). A *p*-value of < 0.05 was considered statistically significant.

## Results and discussion

### Chemistry

The BnTz derivatives (Table 1) were previously synthesised, and their lipophilicity data have been reported (Smit et al. 2019). However, lipophilicity alongside electronegativity was included in the current investigation as parameters susceptible to impact the antitoxoplasmosis activity of the compounds.

**TABLE 1:** Synthesised benzyltriazole derivatives.

Compd.	Structure
FJS-104	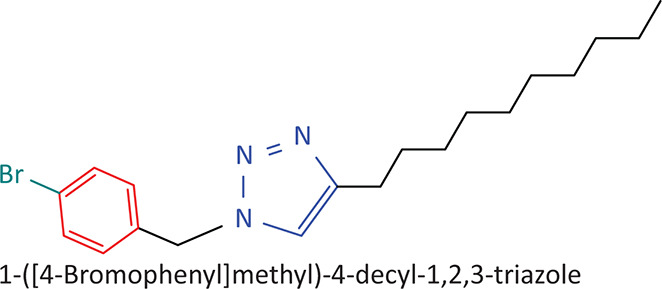
FJS-105	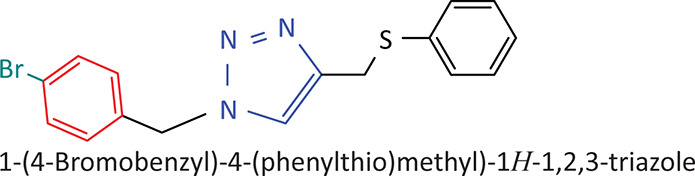
FJS-201	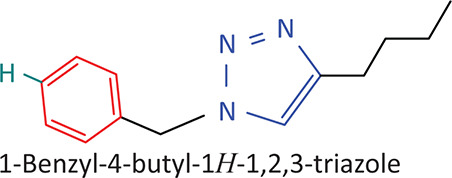
FJS-205	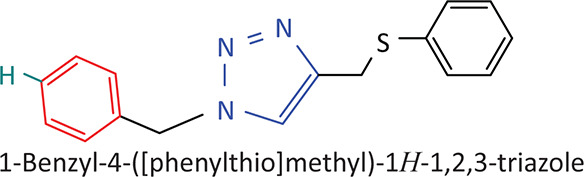
FJS-301	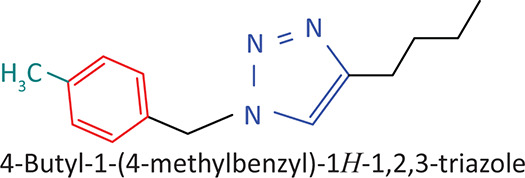
FJS-302	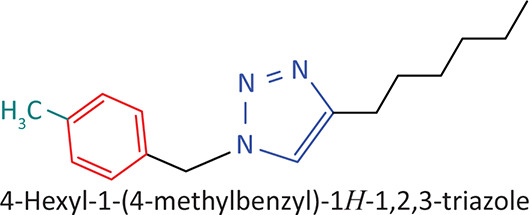
FJS-303	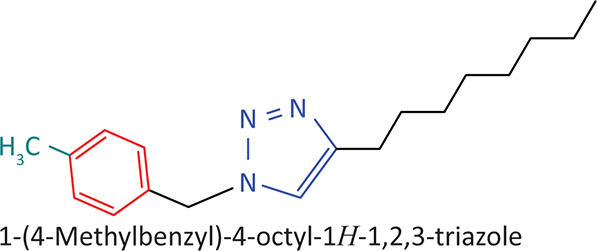
FJS-403	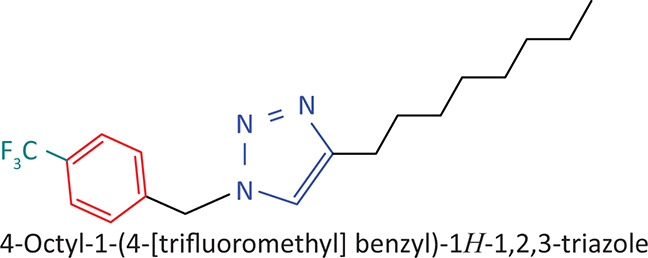
FJS-404	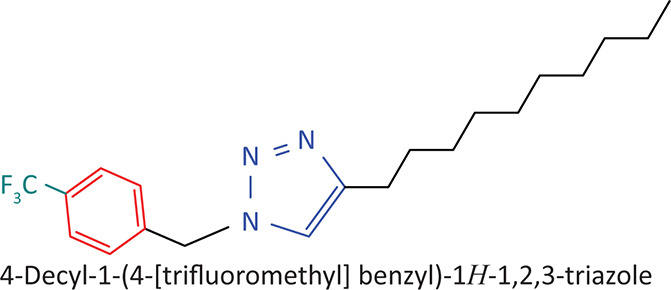
FJS-405	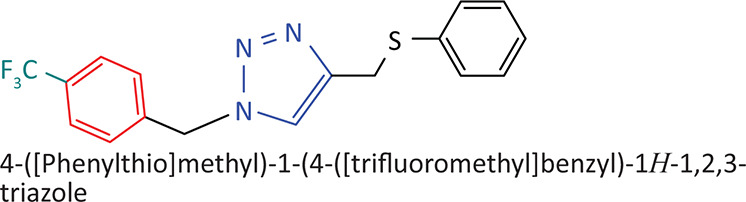

Compd., compound; FJS, Frans J. Smit.

The rationale for introducing the substituents R (H, CH_3_, Br or CF_3_) on the benzene ring was to evaluate the impact the electronic effect might have on the biological activity. The choice of the various electronic groups (Br and CF_3_ – electron withdrawing groups [EWG] with a destabilising effect, CH_3_ – electron donating group [EDG] with stabilising effect) was previously justified (Smit et al. 2019). Similarly, the lipophilic side chains were anchored to the triazole ring to assess the influence of lipophilicity of the pharmacological effect of the compounds. Indeed, the *n*-octanol/water partition coefficient Latency/overhead/gap/Processor (LogP) is a key parameter used for the measurement of the balance between hydrophilicity and lipophilicity. It gives insight into the transport characteristics of a chemical across a biological membrane through passive diffusion (Gombar & Enslein 1996). Partition coefficient values between 1 and 5 are usually targeted, whilst values between 1 and 3 are ideal (Lipinski et al. 2001). For *n*-alkyl substituted compounds, the derivative lipophilic had positive correlation with the chain length (Table 1).

### Screening of chemical compounds for anti-*Toxoplasma* efficacy

The synthesised BnTz derivatives were screened for their *T. gondii* growth inhibitory effects at a final concentration of 50 µg/mL alongside the reference drug SFZ (Sanford et al. 2018). The frontline anti-TB drug isoniazid, herein designated as FJS-INH (Smit et al. 2019), was also included in the screening as a preliminary test in view of future repurposing of this compound.

**FIGURE 1:**
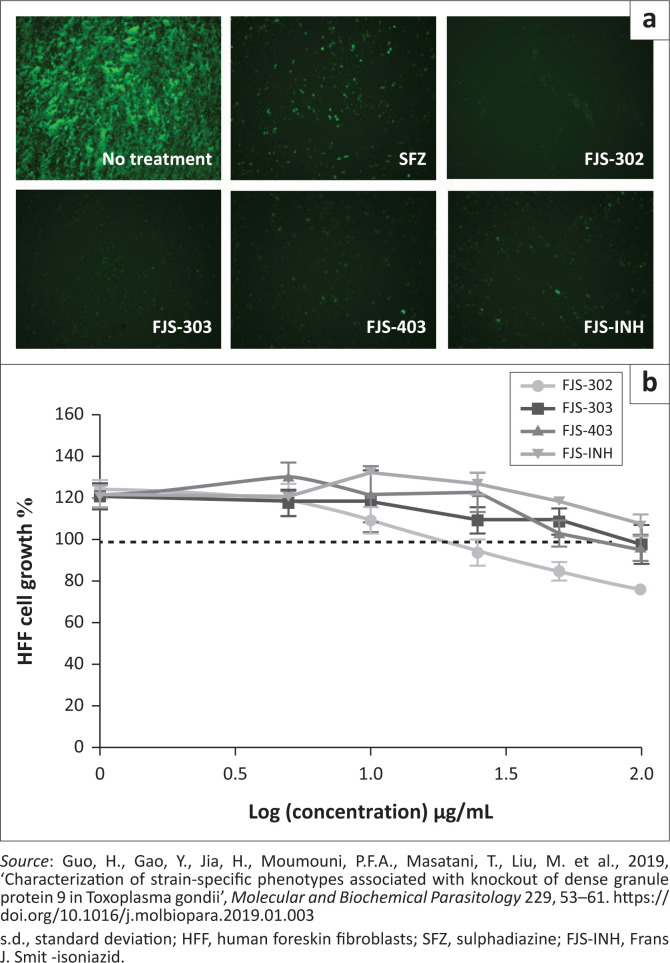
Anti-*Toxoplasma gondii* activity of FJS-302, FJS-303, FJS-403 and FJS-INH on RH-GFP and their cytotoxicity. (a) Representative images of *T. gondii* RH-GFP-infected HFF cells treated for 72 h with sulphadiazine (1 mg/mL), FJS-302 (50 µg/mL), FJS-303 (50 µg/mL), FJS-403 (50 µg/mL) or FJS-INH (50 µg/mL). (b) Cytotoxicity testing of HFF cells after treatment with FJS-302, FJS-303, FJS-403 or FJS-INH at concentrations of 0 µg/mL to 100 µg/mL for 24 h. Data represent the mean values ± s.d. of three independent experiments.

The average inhibition rate of BnTz derivatives was 60% on parasites, whilst FJS-303 showed the highest inhibition with 96%. Three derivatives, namely FJS-302, FJS-303, FJS-403 and the isoniazid FJS-INH, had a parasite growth inhibition rate > 85% (Table 2). Parasite growth at 72 h after infection (Figure 1a) indicates that these three chemical compounds showed better growth inhibitory efficacy than positive control SFZ (1 mg/mL).

**TABLE 2:** Screening of chemical compounds for anti-RH-GFP efficacy.

Compound Entry Code		cLogP†	Anti-toxoplasmosis activity		% viability HFF§	% viability Vero¶
			% Parasite inhibition (mean ± s.d.)‡	IC_50_ (*μ*g/mL)		
1	FJS-104	7.13	62.2 ± 6.0	ND	100.03	102.65
2	FJS-105	4.23	60.5 ± 6.1	ND	100.04	103.31
3	FJS-201	1.95	64.6 ± 6.8	ND	100.02	99.34
4	FJS-205	2.22	60.2 ± 5.1	ND	100.08	100.66
5	FJS-301	3.59	57.7 ± 3.0	ND	96.16	85.43
6	FJS-302	4.65	95.9 ± 4.6	5.6	85.00	86.09
7	FJS-303	5.71	96.3 ± 3.9	6.8	110.35	100.66
8	FJS-403	6.09	91.5 ± 1.7	7.0	103.47	101.97
9	FJS-404	7.15	64.5 ± 1.8	ND	100.05	ND
10	FJS-405	4.25	35.0 ± 5.0	ND	100.23	103.29
11	FJS-INH	−0.67	87.2 ± 4.6	19.8	118.26	98.03
12	SFZ	0.1	73.7 ± 4.6	59.5	105.19	92.11

RH-GFP, expressing green fluorescent protein (GFP) type I strain RH; FJS, Frans J. Smit; FJS-INH, Frans J. Smit -isoniazid; SFZ, sulphadiazine; HFF, human foreskin fibroblasts; cLogP, Latency/overhead/gap/Processor; IC_50_, half-maximal inhibitory concentration; ND, not determined; s.d., standard deviation.

† , Calculated from ChemDraw. Ultra 12;

‡ , Parasite growth inhibition values of parasite-infected cells with test chemical compounds at a concentration of 50 µg/mL and sulphadiazine at a concentration of 1 mg/mL;

§ , HFF cell viability values with test chemical compounds at a concentration of 50 µg/mL and sulphadiazine at a concentration of 1 mg/mL;

¶ , Vero Cell viability values with test chemical compounds at a concentration of 100 µg/mL and sulphadiazine at a concentration of 1 mg/mL.

**FIGURE 2:**
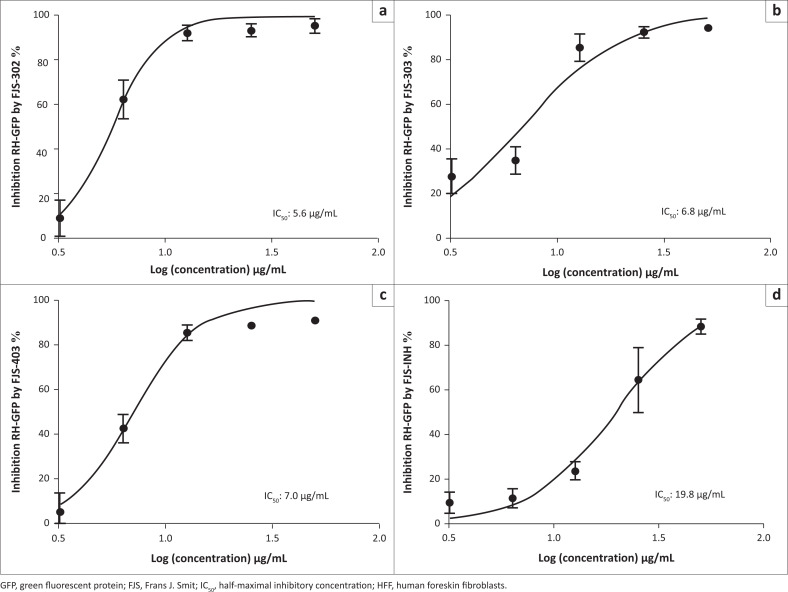
The IC_50_ values of (a) FJS-302, (b) FJS-303, (c) FJS-403 and (d) FJS-INH on *Toxoplasma gondii* RH-GFP. The RH-GFP-infected HFF cells were treated with the given chemical compounds for 72 h at different concentrations from 3.125 µg/mL to 50 µg/mL. Data represent the mean values ± s.d. of three independent experiments.

The effect of FJS-302, FJS-303, FJS-403 and FJS-INH on host cell viability was examined, and no significant suppression on host cell growth was observed, even at 100 µg/mL (Figure 1b), suggesting that the effect of these BnTz compounds on RH-GFP growth was not a consequence of host cell cytotoxicity. Consequently, the parasite inhibitory effects of the screened compounds at different concentrations were evaluated. The IC_50_ values of FJS-302, FJS-303 and FJS-403 were 5.6 µg/mL, 6.8 µg/mL and 7.0 µg/mL, respectively (Figure 2), whilst the IC_50_ value of SFZ was 59.5 µg/mL (Table 2). The inhibition ratios of FJS-104 to FJS-301, FJS-404 and FJS-405 were lower than that of SFZ, so their IC_50_ value was not evaluated.

De Oliveira et al. (2009) reported that the IC_50_ value of SFZ on RH strain was 70 µg/mL, and the viability of HFF cells decreased by 28% in the presence of 200 µg/mL of SFZ. Thus, FJS-302, FJS-303, FJS-403 and FJS-INH should be more effective at controlling the growth of *T. gondii in vitro* than SFZ.

The electronic effect had limited impact on the activity of the derivatives. Indeed, in comparing the IC_50_ values of the active compounds, it can be seen (Table 1) that there are no significant differences amongst FJS-302 (IC_50_ 5.6 µg/mL), FJS-303 (IC_50_ 6.8 µg/mL) and FJS-403 (IC_50_ 7.0 µg/mL). Similarly, the lipophilicity had marginal influence on compound activity. Indeed, based on the cLogP and IC_50_ values, the three active BnTz derivatives had comparable lipophilicity and activities against *T. gondii*. However, a realistic conclusion on structure–activity relationship of the compounds can only be drawn through investigation of a wider series of BnTz derivatives.

Another interesting finding of this study is the activity of isoniazid. *In vitro* inhibition of this mainstay anti-TB drug against *T. gondii* has previously been reported (Sanford et al. 2018). In this study, FJS-INH was efficient on *T. gondii* growth inhibition, although threefold less potent than the leading BnTz derivative. The activity of FJS-INH (IC_50_ of 19.8 µg/mL) may be suggestive of the potential of alternative use as antitoxoplasmosis agent. Thus, further optimisation through investigation of novel derivatives of FJS-INH for treatment of toxoplasmosis may be worth it.

### Effects of chemical compounds on parasite invasion and *in vitro* replication

**FIGURE 3:**
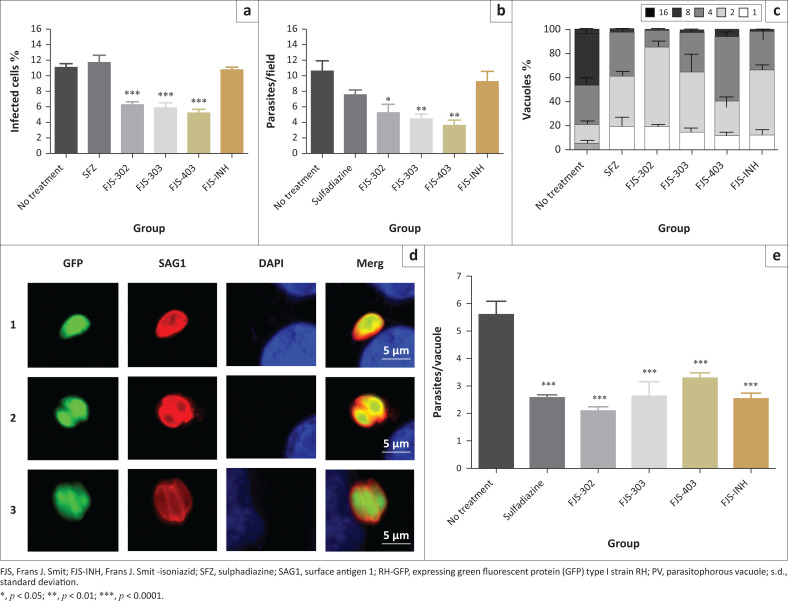
Effects of FJS-302, FJS-303, FJS-403 and FJS-INH on *Toxoplasma gondii* invasion and replication. FJS-302 (10 mg/mL), FJS-303 (12.5 mg/mL), FJS-403 (12.5 mg/mL), FJS-INH (50 mg/mL) and sulphadiazine (1 mg/mL). (a) The percentage of infected cells was evaluated by counting the number of SAG1-positive Vero cells per 100 Vero cells by using IFAT. Each bar represents the mean ± s.d. of three independent experiments. (b) The number of parasites per field was measured by using a microscope. At least 10 fields were randomly observed in each group. Each bar represents the mean ± s.d. of three independent experiments. (c) Representative images of *T. gondii* RH-GFP replication in Vero cells. (d) The number of parasites in parasitophorous vacuoles was measured by counting the number of SAG1-positive parasites per parasitophorous vacuole. Each bar represents the mean ± s.d. of three independent experiments. (e) The average number of parasites per parasitophorous vacuole. Each bar represents the mean ± s.d. of three independent experiments.

To test whether these compounds are active on inhibition of parasite invasion, purified parasites were pretreated with FJS-302 (10 µg/mL), FJS-303 (12.5 µg/mL), FJS-403 (12.5 µg/mL), FJS-INH (50 µg/mL) or 1 mg/mL of SFZ before the infection of Vero cells. Infection rate of non-treated cell was 11.3% ± 0.6%. Pretreatment with FJS-302, FJS-303 and FJS-403 significantly decreased the infection rates to 6.2% ± 0.5%, 5.8% ± 0.5% and 5.2% ± 0.7%, respectively, whilst pretreatment with FJS-INH and SFZ showed 10.7% ± 0.5% and 11.7% ± 1.3% infection rates (Figure 3a). At the same time, the number of parasites per field after pretreatment with FJS-302, FJS-303 or FJS-403 was also significantly reduced (Figure 3b), suggesting that these BnTz derivatives were effective in inhibiting the invasion of extracellular parasites.

To examine the effect of the four chemical compounds on parasite replication, RH-GFP-infected Vero cells were treated with the same concentration of chemical compounds or SFZ. The number of tachyzoites per PV was counted (Figure 3c). The percentage of PV containing four or more tachyzoites was 78.7% in no treatment group whilst this percentage was lower in the groups treated with chemical compounds or SFZ (Figure 3d). In addition, the average number of tachyzoites per PV after treatment was significantly reduced compared with the no treatment group (Figure 3e). These data indicate that all four chemical compounds inhibited parasite replication.

The different life stages of *T. gondii* are vital for the parasite survival in intermediate and definitive hosts; however, the tachyzoite is the rapidly multiplying stage of the parasite (Maenz et al. 2014). Through their rapid replication, tachyzoites can damage host tissue. FJS-302, FJS-303 and FJS-403 were effective against extra- and intracellular parasites, whilst SFZ affected only intracellular parasite.

### Effects of chemical compounds on bradyzoite induction

**FIGURE 4:**
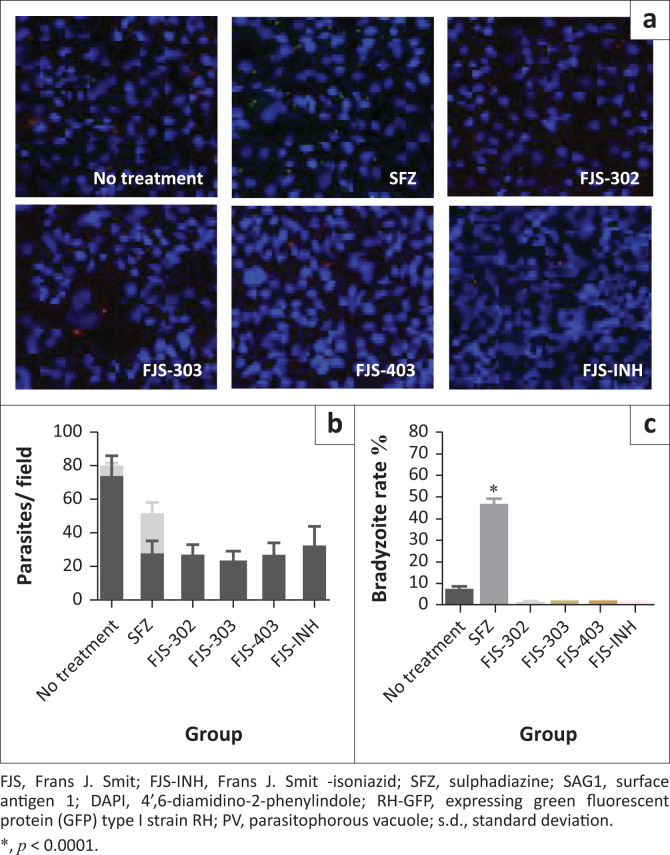
Effects of FJS-302, FJS-303, FJS-403 and FJS-INH on *Toxoplasma gondii* bradyzoite induction. (a) Representative images of the sulphadiazine (1 mg/mL), FJS-302 (50 µg/mL), FJS-303 (50 µg/mL), FJS-403 (50 µg/mL) or FJS-INH (50 µg/mL) treated wells. Red, SAG1; Green, GFP; Blue, DAPI. (b) GFP-positive parasitophorous vacuole (bradyzoite) number and anti-SAG1 positive parasitophorous vacuole (tachyzoite) number were counted. (c) GFP-positive parasitophorous vacuole rate was calculated by counting total parasitophorous vacuole and GFP-positive parasitophorous vacuole per image. Each bar represents the mean ± s.d. of three independent experiments.

To determine whether the BnTz derivatives-treated tachyzoite could induce bradyzoite differentiation, we assessed the bradyzoites ratio of PruΔku80Δhxgprt, a *T. gondii* strain that can undergo spontaneous bradyzoite differentiation (Murata et al. 2017) when parasites are incubated at their most effective concentration. Indirect fluorescent antibody test was performed to determine the number of tachyzoites and bradyzoites after 48 h of treatment of the infected cells with specific concentration of the four chemical compounds and SFZ (Figure 4a and 4b). Parasite growth was estimated by the total number of tachyzoite and bradyzoite in each field (Figure 4b). All tested compounds reduced the total parasite and tachyzoite numbers. However, bradyzoite number in the SFZ group (23.7 ± 6.0) was higher than that of the no treatment group (6.5 ± 1.5). Regarding the bradyzoite rate, SFZ significantly induced bradyzoite differentiation (46.6% ± 7.9%), whereas none of the four chemical compounds induced a bradyzoite differentiation. Bradyzoite differentiation in compound-treated group was comparable to the no treatment group (8.2% ± 1.9%) (Figure 4c). This demonstrates that the four chemical compounds are not selective for tachyzoites and have no bradyzoite-inducing effect.

Whilst some therapy exists for the treatment of acute *T. gondii* infection, it is necessary to develop new therapeutic agents that are active against both acute and chronic infection and have mild side effects and low toxicity on host cells. PruΔku80Δhxgprt with a bradyzoite reporter is useful for the evaluation of chemical compounds for their effects on both tachyzoites and bradyzoites (Murata et al. 2017). Here, FJS-302, FJS-303, FJS-403 and FJS-INH had significant antitachyzoite activity and a degree of non-inducing effect on bradyzoites. Agents that can eliminate tachyzoites and bradyzoites may be used to treat both acute and chronic infection. Several compounds that target the replication of bradyzoites have been identified (Doggett et al. 2014; Murata et al. 2017). The effects of these chemical compounds against *T. gondii in vivo* should also be evaluated.

## Conclusion

In this study, a series of previously synthesised BnTz derivatives, FJS-302, FJS-303, FJS-403 and FJS-INH inhibited tachyzoite growth. FJS-302, FJS-303 and FJS-403 were effective against extra- and intracellular parasites, whilst the current recommended drug SFZ affected only intracellular parasite. In addition, SFZ induced bradyzoite differentiation, whereas FJS-302, FJS-303 and FJS-403 did not increase the bradyzoite number. These results indicate that FJS-302, FJS-303 and FJS-403 have the potential to act as a viable source of antiparasitic therapeutic agents.
